# Oleanolic Acid Enhances Mesenchymal Stromal Cell Osteogenic Potential by Inhibition of Notch Signaling

**DOI:** 10.1038/s41598-017-07633-7

**Published:** 2017-08-01

**Authors:** Bing Shu, Yongjian Zhao, Yongjun Wang, Guangxi Wang, Xifu Shang, Michael Britt, Margaret Olmedo, Marjorie Chelly, Massimo Max Morandi, Shane Barton, Yufeng Dong

**Affiliations:** 1 Longhua Hospital and Key Laboratory of Ministry of Education of China, Shanghai University of Traditional Chinese Medicine, Shanghai, China; 2 Department of Orthopedic Surgery, LSU Health Sciences Center, Shreveport, LA USA; 3 Department of Orthopedic Surgery, Anhui Provincial Hospital, Hefei, Anhui China

## Abstract

Oleanolic acid (OA), a pentacyclic triterpenoid, has been shown to modulate multiple signaling pathways in a variety of cell linages. But the mechanisms underlying OA-mediated mesenchymal stromal cell (MSC) osteogenic differentiation are not known. In this study, we examined effects of OA on cell viability, osteogenic differentiation in MSCs, and the involvement of Notch and BMP signaling. OA induced bone marrow derived MSC differentiation towards osteoprogenitor cells and inhibited Notch signaling in a dose dependent manner. Constitutive activation of Notch signaling fully blocked OA induced MSC osteogenic differentiation. The expression level of early osteogenic marker genes, ALP, Runx2, and type I collagen, which play a critical role in MSC to osteoblast transition and servers as a downstream target of BMP signaling, was significantly induced by OA. Furthermore, BMP2 mediated MSC osteogenic differentiation was significantly enhance by OA treatment, indicating a synergistic effect between BMP2 and OA. Our results suggest that OA is a promising bioactive agent for bone tissue regeneration, and inhibition of Notch signaling is required for its osteogenic effects on MSCs.

## Introduction

Bone fracture healing and large bone defect healing are a large societal problem that is getting worse due to the increase of patients with bone loss caused by car accident, battlefield injury, and tumor resection^1, 2^. To date, surgery remains the best choice of treatment. More than one million traumatic fractures require surgical intervention in the US each year and approximately 10% result in delayed union or non-union due to reduced local mesenchymal stromal cell (MSC) osteogenic differentiation. Particularly in old people or in patients with diabetes, fractures take a long time to heal or may never heal leading to long-term disability and/or amputation^3–5^. Although growth factor bone morphogenetic protein 2 (BMP2) has been clinically used to enhance MSC differentiation toward bone forming cells during spine fusion surgery, adverse effects still remain, including osteoclast activation, life-threatening inflammatory swelling, and adipogenesis, when high dose of BMP2 is used^6–8^. To develop an alternative bioactive agents to replace BMP2 or in combination to reduce the dosage and the cost of BMP2, become necessary.

As a natural pentacyclic triterpenoid, oleanolic acid [(3β)-3-hydroxyolean-12-en-28-oic acid] (OA) is an aglycone of many saponins, and one of the most important compounds with bioactivity in medicinal herbs^9^. OA exists widely in food products (vegetable oils) and could be extracted from the leaves and roots of many plant species, such as *Olea europaea*, *Viscum album* L., and *Aralia chinensis* l^10, 11^. OA exhibits various biological properties, including anti-inflammatory, antidiabetic, and hepatoprotective effects^12, 13^. After absorption, OA is mainly distributed and transformed in the liver. It has been proven that OA protects mice from various hepatotoxicants, such as carbon tetrachloride, acetaminophen, bromobenzene, and thioacetamide, and OA displayed no significant cytotoxicity to normal cells^14^. Indeed, OA has been used for over two decades in Chinese medicine for the treatment of liver disorders, such as viral hepatitis^15^. In recent years, accumulating evidence has shown OA exhibits metabolic activity in many cellular processes, including apoptosis^16^, cell cycle arrest, and differentiation^17^. Studies by Bian, *et al*.^18^ showed that oleanolic acid exerts an osteoprotective effect in ovariectomy-induced osteoporotic rats and stimulates the osteoblastic differentiation of bone mesenchymal stem cells *in vitro*. In contrast, other studies indicate that osteoprotective effect of oleanolic acid is due to inhibited RANKL-mediated osteoclastogenesis via PLCγ2-Ca2+ -NFATc1 signaling^19^. However, the effects of OA on stem cell osteogenic differentiation have not been investigated in detail, and the underlying mechanisms responsible for bone forming effect of OA are still unknown. Various signaling factors have been implicated in the regulation of MSC osteoblastic differentiation. Recently, our group along with others have identified the Notch pathway as an important inducer of MSC proliferation, and as an inhibitor of MSC differentiation during mouse limb-bud and postnatal bone development^20^. Activation of Notch signaling induces cleavage and release of the Notch intracellular domain (NICD), which translocates from the cell surface into the nucleus to activate target gene expression of Hes1 via a NICD-RBPJK-MAML transcriptional complex^21^.

In this study, we first studied the effect of OA on Notch signaling-mediated MSC osteogenic differentiation, and then determined the effect of OA on BMP-induced osteogenesis.

## Materials and Methods

### Cell culture and treatment

The human MSCs was purchased from Lonza. Oleanolic acid was obtained from Sigma-Aldrich (Aldrich-5504). A 100 mM solution of OA was prepared in dimethyl sulfoxide (DMSO), and all test concentrations were prepared by diluting the stock solution in cell culture medium. Recombinant human BMP2 was purchased from R&D. The cells were cultured in DMEM supplemented with 10% fetal bovine serum (Gibco BRL, Rockville, MD). After expansion, cells were exposed to media contains OA and/or BMP2 for certain days. Since the half-life of OA is around eight to 16 hours^22^, we decided to change media once a day for up to seven days to induce MSC differentiation. At the end, cells were harvested at different time points for analyses as described below.

### Luciferase assay

80% confluence MSCs were transfected with Notch responsive RBPJ-Luc and SV40-Renilla-Luc (Promega) in the presence of Lipofectamine 2000 (Invitrogen) followed by OA treatment. At 24 hours after transfection and treatment, lysates were analyzed with a Dual Luciferase Assay Kit (Promega).

### Cell viability assay

MSC viability was measured using MTT (3-(4, 5-dimethylthiazole-2-yl)-2, 5-diphenyl tetrazolium bromide) based *in vitro* toxicology assay kit (Sigma, St. Louis, MO, USA). Briefly, cells were seeded in 96-well plates. After growing into 50~60% confluence, cells were exposed to various concentrations of OA for 12 hours, then the supernatant was discarded, and cells were treated with 0.5 mg/ml MTT staining solution for another four hours. After incubation, the supernatant was removed and 50 μl of solubilization buffer provided by the Sigma kit with 0.5% DMSO was added. DMSO was added to ensure total solubility of the formazan crystals. Plates were shaken for two minutes, and the absorbance recorded at 590 nm on an automated microtiter plate reader. The percent viability was expressed as a percentage of that in the vehicle control (with subtraction of background absorbance).

### MSC osteogenic differentiation assay

Osteogenic differentiation assays of MSCs were performed using the Osteogenic differentiation bulletKit® (Lonza). MSCs were cultured at 200,000 cells/well in 6-well plates to confluence. Stem cell growth media was then replaced with osteogenic media supplied with or without OA and BMP2. After seven days of culture, alkaline phosphatase (ALP) staining, RNA, and protein isolation were performed as described in our published protocols^20^.

### Real time RT-PCR

cDNA was synthesized from 1 μg total RNA using the SuperScript III reverse transcriptase kit (Invitrogen) in a final volume of 20 μl. Primers were designed with the IDT SCI primer design tool (Integrated DNA Technologies, San Diego, California). RT-PCR experiments were performed with a Bio-Rad C1000 thermal cycler (Bio-Rad, Hercules, CA), and real-time PCR experiments were performed with an ABI prism 7500 (Applied Biosystems, Grand Island, NY) in triplicate. Sequence for each primer pair were: Hes1, forward primer 5′-TTCCTCCTCCCCGGTGGCTG-3′, reverse primer 5′-TGCCCTTCGCCTCTTCTCCA-3′; ALP, forward primer 5′-GGGCATTGTGACTACCACTC-3′, reverse primer 5′-AGTCAGGTTGTT CCGATTCA-3′; Runx2, forward primer 5′-CACTGCCACCTCTGACTTCT-3′, reverse primer 5′-CACCATCATTCTGGTTAGGC-3′; Osteocalcin (OC), forward primer 5′-TGGCCATGCTGACTGCAGCC-3′, reverse primer 5′-TGGGTAGGCGTCCCCCATGG-3′; Osteopontin (OPN), forward primer 5′-AAGGAACCAAAGCATCAAGAATTAG-3′, reverse primer 5′-AGATGTCATCAGGCAGCTTGAC-3′; Type I collagen (Col1a1), forward primer 5′-GTTTGGCCTGAAGCAGAGAC-3′, reverse primer 5′-TCTAAATGGGCCACTTCCAC-3′; β-actin, forward primer 5′-ACCACAGTCCATGCCATCAC-3′; reverse primer 5′-TCCACCACCC TGTTGCTGTA-3′. Samples were analyzed in triplicate, and the raw data consisted of PCR cycle numbers required to reach a fluorescence threshold level. The relative expression level of target genes was normalized to β-actin gene^23^.

### Lentivirus-mediated Notch activation

cDNAs encoding human Notch1-NICD was cloned into the EF.v-CMV lentiviral vector and sequence verified. Lentivirus production and concentration was performed as previously described^24^. Briefly, VSV.G-pseudotyped recombinant lentiviruses were produced by transient transfection of the transducing vector into 293 T cells, along with two packaging vectors: pMD.G, a VSV.G envelope-expressing plasmid, and pCMVΔR8.91 (Invitrogen, Carlsbad, CA, USA), containing the HIV-1 *gag*/*pol*, *tat*, and *rev* genes (1.5 μg: 2.0 μg: 0.5 μg ratio of these three vectors). Viral supernatants were collected at 24, 48 and 72 hours after transfection, and concentrated using filtration columns (Centricon Plus-20, molecular weight cutoff = 100 kDa; Millipore, Bedford, MA, USA). For lentiviral infection, 10 ml high-density (10,000 cell/ml) MSCs were seeded in 6-well plates and incubated for two hours at 37 °C prior to be added with osteogenic medium supplied with NICD1 lentivirus and 8 μg/ml polybrene. GFP-lentivirus was used in this experiment as a control. The infected MSC cultures were harvested at day seven for ALP staining, protein and RNA isolation.

### Western blot analysis

MSCs cultured with or without OA and BMP2 treatment were lysed in RIPA buffer (10 mM Tris–HCl, 1 mM EDTA, 1% sodium dodecyl sulfate [SDS], 1% NP-40, 1: 100 proteinase inhibitor cocktail, 50 mM β-glycerophosphate, 50 mM sodium fluoride). The samples were separated on a 10% SDS polyacrylamide gel and transferred to polyvinylidene difluoride (PVDF) membranes with a semi-dry transfer apparatus (Bio-Rad). The membranes were blotted with 5% dehydrated milk for two hours, and then, incubated with primary antibodies overnight. The immune complexes were incubated with horseradish peroxidase-conjugated anti-rabbit or anti-mouse IgG (Promega, USA), and visualized with SuperSignal reagents (Pierce, USA). Primary polyclonal antibodies against NICD1 and phosphor-smad1/5/8 (Cell Signaling, USA) were used. We also used a primary monoclonal antibody to detect the housekeeping protein, β-actin (Sigma-Aldrich, USA).

### *In vivo* ectopic bone-formation assay

All animal studies were reviewed and approved by the Animal Ethics Committee of Louisiana State University Health Sciences Center. 1.0 × 10^6^ total MSCs were re-suspended in 80 μl of MSC growth media contains 20 *µ*M OA and gently mixed with 50 mg of hydroxyapatite (HA) powder (Himed). The composites were then centrifuged for two minutes at 200 rpm to form a MSC/HA pellet. For ectopic bone formation surgery, ten 8-week-old NOD.CB17^−Prkdcscid/J^ mice (Charles River) (up to 2 implants for each animal) were anesthetized and the dorsal skin was cleaned with 70% ethanol. Two incisions of ~1 cm in length were performed on the opposite flanks of the back, and then a pocket was formed by blunt dissection. Finally the MSC/HA pellets were implanted. Six weeks post-surgery, transplanted BMSC/HA pellets were harvested for gross observation and Masson’s trichrome staining. Immunohistochemistry (IHC) staining was performed using anti-osteopontin antibody (7C5H12, Abcam, USA) according to our published protocols^23^. All animal work was performed in accordance with institution approved guidelines.

### Statistical Analysis

All experiments were repeated at least three times independently. Data was presented as mean ± s.d. Statistical significance among the groups was assessed with one-way ANOVA. The level of significance was p < 0.05.

## Results

### OA induces osteogenic differentiation of MSCs while inhibits Notch signaling in a dose-dependent manner

**Figure 1:**
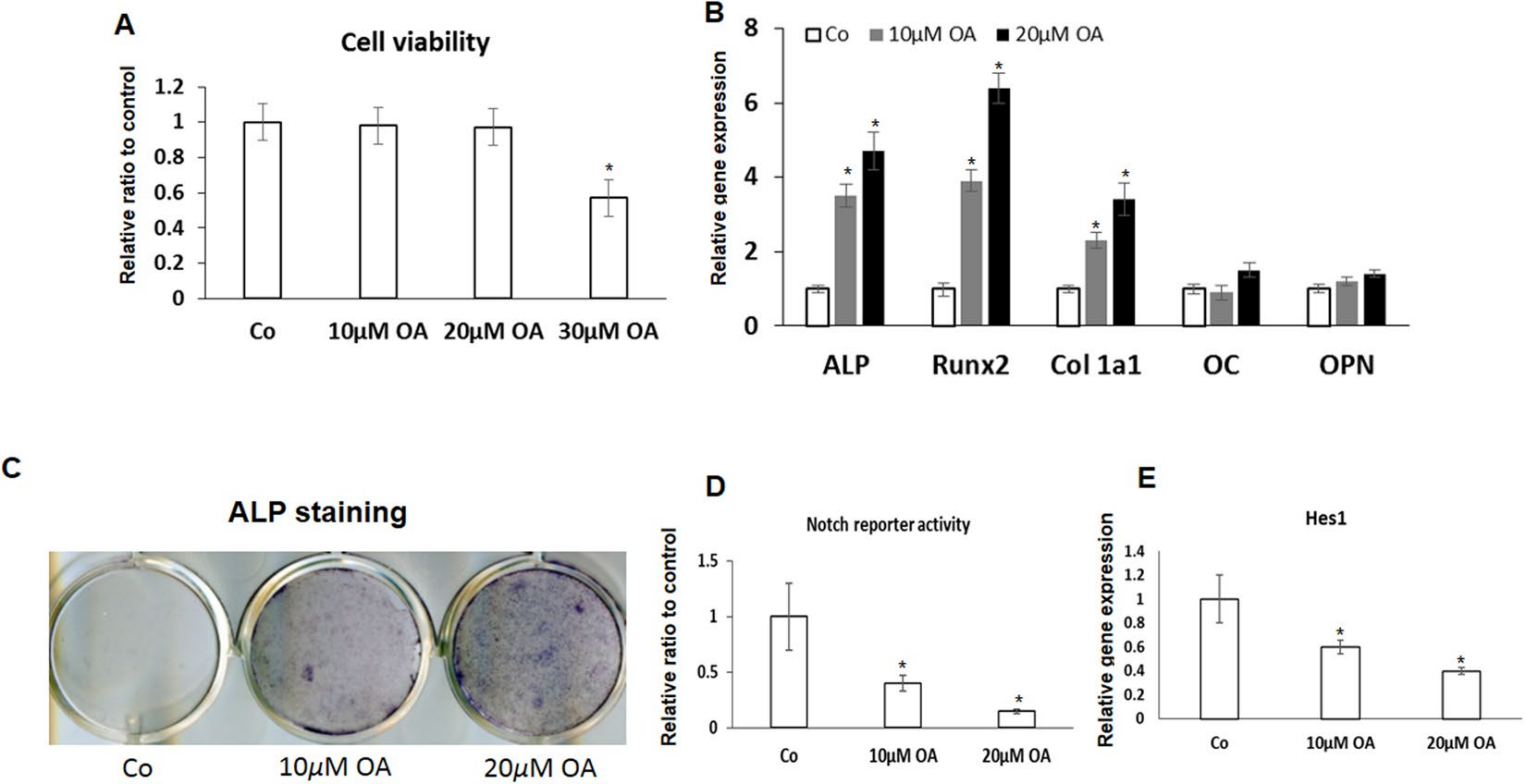
Inhibition of Notch signaling by OA enhances inducted MSCs osteogenic differentiation. (**A**) Cell viability assays showed a significant increase of cell death was only observed in high dose of OA (30 µM) treatment for 12 hours. (**B**) Quantification of gene expression of osteogenic markers indicates a significant increase (p < 0.05) of early stage markers of ALP, Runx2, and type I collagen (Col1a1); and no significant change was observed in expression of later markers Osteocalcin (OC) and Osteopontin (OPN). Data is the means ± s.d. of three independent experiments performed in duplicate and the shRNA-lentivirus control (Co) gene expression level was set at 1. (*p < 0.05 compared with control) (**C**) An increase in osteogenic nodule formation was observed in OA treated MSCs at day 7, with maximal staining of OA treatment at 20 *µ*M. Scale bars, 100 µm. (**D**) Luciferase assays showed a significant decrease of Notch responsive reporter activity in OA treated MSCs in a dose dependent manner. Data is the means ± s.d. of three independent experiments performed in duplicate and all the results were normalized to internal control (*p < 0.05 compared with control MSCs (Co) without OA treatment). (**E**) Real time PCR data showed a significant decrease of Notch target gene Hes1 expression at day 7 following OA treatment compared to DMSO treated control MSCs (Co).

In order for bones to regenerate, specific mesenchymal stem cells (MSCs) surrounding the injured site have to be first differentiate into osteoprogenitor cells, and then become functional osteoblasts. To examine the effects of OA on the cell viability in MSCs, we performed the *in vitro* cell viability assays, after 12 hours treatment of OA in three different doses, a significant cell death was observed in cells with the dose of 30 µM, and no cell death was noticed in cultures with low doses of 10 µM and 20 µM treatment (Fig. 1A). Therefore, we selected to use 10 *µ*M and 20 *µ*M of OA in subsequent experiment. MSC osteogenic differentiation was further observed in osteogenic differentiation cultures following treatment with two different doses of OA for seven days. Cultures from OA treated MSCs exhibited enhanced expression of early osteogenic markers, *Col1a1*, *ALP*, and *Runx2*, as compared to cultures from control MSCs treated with DMSO (Fig. 1B). Interestingly, the later osteogenic markers *OC* and *OPN* were remain unchanged (Fig. 1B), indicating OA mainly functions at the onset stage of MSC osteogenic differentiation. Finally, ALP staining in cultures indicated a dose dependent increase of MSC osteogenic differentiation in OA treated MSCs by showing a stronger staining (Fig. 1C) further confirmed this enhanced osteogenic differentiation in OA treated MSCs.

Accumulating evidences indicate that Notch signaling plays a key role in regulating cell growth and differentiation during development. We have previously identified the RBPjk-dependent Notch pathway as an important inducer of MSC proliferation, and an inhibitor of MSC differentiation during mouse limb-bud and postnatal bone development^20^. In this study, we further tested whether Notch signaling is also involved in OA-mediated osteogenesis. To do so, MSCs were first transfected with the RBPjk-dependent NOTCH-responsive luciferase reporter, and then cultured in medium contains 0, 10uM and 20 *µ*M OA for 48 hours. The results revealed that OA inhibited Notch-responsive luciferase activity dose dependently in MSCs (Fig. 1D). Moreover, down-regulation of Notch target gene Hes1 expression was also observed in OA treated MSCs (Fig. 1E), which further confirmed the inhibitory effect of OA on Notch signaling. These results suggested that OA had the ability to promote osteogenesis while preserving cell viability at the concentration of 20 *µ*M, so we decided to use 20 *µ*M OA treatment for our subsequent experiments.

### Constitutive Notch activation blocks OA-induced osteogenic differentiation

**Figure 2:**
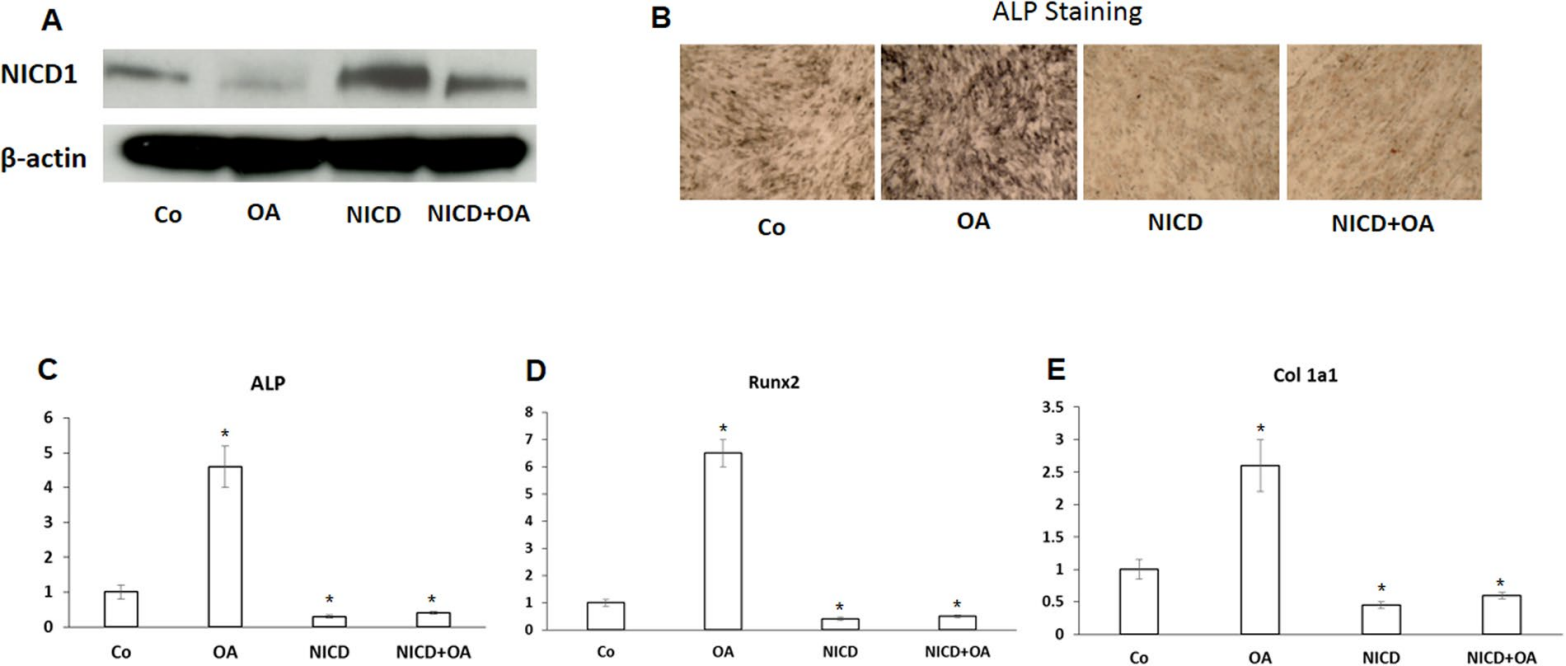
Notch signaling blocks OA-induced osteogenesis in MSC culture. NICD1 lentivirus infected MSCs were treated with OA before being harvested for western blot, ALP staining, and RT-PCR analysis. (**A**) Western Blot showed a significant increase of NICD protein expression at day 3 following NICD1 lentiviral infection. Full-length blots/gels are presented in Supplementary Figure S1. (**B**) Overexpression of NICD1 resulted in decreased ALP staining at day 7, compared to GFP-lentirvirus infected controls (Co). (Magnification: ×5). (**C**,**D**,**E**) Real time PCR data showed a significant increase of ALP, Runx2, Col1a1 expression at day 7 following OA treatment and these induced expression was significantly reduced by NICD1 lentiviral infection compared to GFP-lentirvirus infected controls (Co). Data is the means ± s.d. of three independent experiments performed in duplicate and the control gene expression level at day 7 was set at 1 (*p < 0.05 compared with control).

Although we observed a reduction of Notch signaling activity in OA treated MSCs, whether Notch inhibition is specifically required for OA mediated osteogenesis is unknown. To determine whether Notch signaling is the key player responsible for OA effect on MSC osteogenesis, we activated Notch signaling in MSCs by overexpressing Notch intracellular domain (NICD1) with lentivirus transduction. The western blot data in Fig. 2A showed that the expression of NICD was significantly reduced by OA treatment, and constitutive overexpression of NICD1 by lentivirus significantly induced Notch signaling in both OA treated and untreated MSCs. Next, we performed ALP staining in NICD1 expressing MSCs. The image in Fig. 2B clearly shows a weak staining in NICD1 infected MSCs when compared to control cells. More importantly, OA-induced ALP staining was totally blocked by overexpression of NICD1 suggesting that down regulation of Notch signaling is required for OA induced MSC osteogenic differentiation. The expression of early osteogenic markers, such as ALP, Runx2 and Col1a1, was also examined by RT–PCR. As expected, the expression of these genes was significantly reduced in both Notch activated MSCs with or without treatment of OA when compared to either control or OA treated MSCs (Fig. 2C,D,E).

### OA enhances BMP2-induced MSC osteogenic differentiation

**Figure 3:**
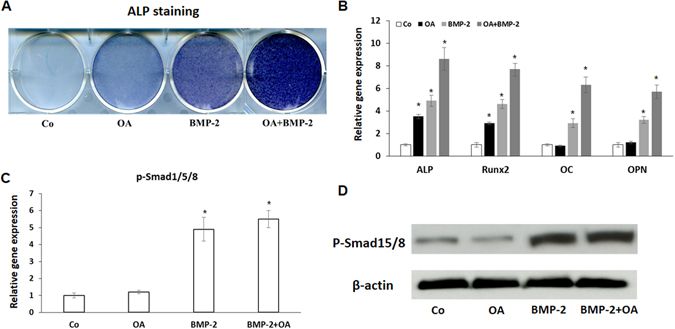
Synergistic effect of OA and BMP2 on MSC osteogenic differentiation. (**A**) ALP staining showed a significant increase of ALP activity in MSCs treated with either OA or BMP2, and this increase is further enhanced by the combined treatment at day 7. (**B**) Real time RT-PCR analysis reveals that expression of both early and later osteogenic markers (ALP, Runx2, OC, OPN) were significantly enhanced by combined treatment with OA and BMP2 at day 7 when compared with either OA or BMP2 alone treated MSCs. PCR data is means ± s.d. of three independent experiments performed in duplicate and all the results were normalized to control (*p < 0.05 compared with control MSCs (Co) without treatment). (**C**) RT-PCR data shows treatment of OA in MSCs did not cause a significant change in gene expression of phosphor-smad1/5/8 at day 7. Data is the means ± s.d. of three independent experiments. (*p < 0.05 compared with control MSCs). (**D**) Western Blot shows phosphor-smad1/5/8 (p-smad1/5/8) protein levels were not affected by OA treatment in MSC culture at day 7. β-actin was used as a loading control. Full-length blots/gels are presented in Supplementary Figure S1.

BMP2 is well known for its osteogenic properties and is used clinically in the spine fusion surgery^25^. Since these osteogenic markers induced by OA (Fig. 1) are also regulated by BMP signaling, we further investigated the effects of OA on BMP2 stimulated osteogenesis. To do so, MSCs were first cultured with OA (20 *µ*M) and/or BMP2 (200 ng/ml) for seven days in osteogenic medium, and then harvested for ALP staining and total RNA isolation. ALP staining image in Fig. 3A showed that ALP activity was significantly enhanced by the combination of OA and BMP2 compared with groups treated with either OA or BMP2 alone. Real-time PCR Results further confirmed that treatment with a combination of OA and BMP2 synergistically enhanced the gene expression of osteogenic markers, ALP, Col1a1, Runx2, OC, and OPN (Fig. 2A). As phospho-smad1/5/8 (p-smad1/5/8) is the key downstream factor in BMP signaling, we next measured the expression of p-smad1/5/8 in OA-mediated MSC osteogenic differentiation. As shown in Fig. 3C, BMP2 treatment significantly increased gene expression of p-smad1/5/8 in MSCs. In contrast, OA treatment did not show any effects on the expression of p-smad1/5/8 in both MSCs with or without BMP2 treatment. To confirm these results, we further isolated total protein from BMP2 and/or OA treated MSCs. Consistent with RT-PCR data, the western blot data clearly showed that no significant difference of p-smad1/5/8 expression was observed between control and OA treated MSCs; thus, suggesting OA induced MSC osteogenic differentiation in a BMP signaling independent manner.

### OA enhances BMP2-mediated ectopic bone formation

**Figure 4:**
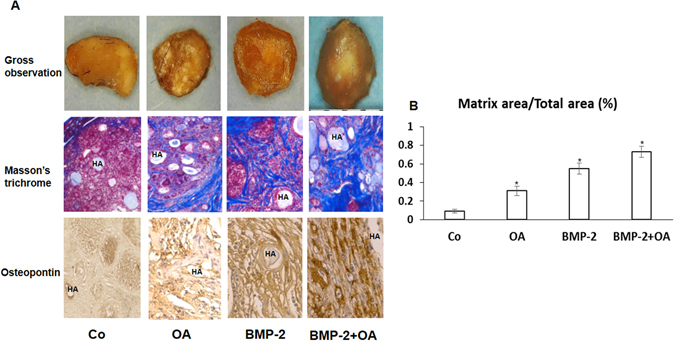
OA treatment enhances MSC ectopic bone formation *in vivo*. (**A**) Top panel: Gross observation of transplanted MSC/HA pellet immediately after fixation. Middle panel: Masson’s Trichrome staining of tissue sections from the ectopic ossicle formation samples at 6 weeks after implanted with MSCs that were previously mixed with OA and/or BMP2. Blue staining denotes a Col1a1 rich bone-like matrix in the ossicles. Low panel: Immunohistochemistry staining of osteopontin in transplanted MSC/HA pellet. (**B**) Areas of bone-like matrix (blue) staining on Masson’s Trichrome stained sections were quantified vs total area using ImageJ for all groups (*n* = 3; magnification, ×20).

To further confirm the capacity of OA induced osteogenesis *in vivo*, we performed ectopic ossicle assays using 8-week old nude mice. MSCs were first dissolved in PBS containing 20 *µ*M OA, then mixed with HA ceramic powder, and centrifuged into MSC/HA pellets prior to subcutaneous transplantation into nude mice. After six weeks of growth and differentiation, we harvested the pellets for gross observation, Masson’s trichrome and immunohistochemistry staining (Fig. 4A). Gross observation of harvested ectopic bone formation pellet showed a round-shaped pellet with 2–3 mm in diameter from each groups. No significant difference was noticed regarding the weight, size and stiffness of these pellets in four different groups. As we expected, approximately 4-fold increase of type I collagen bone matrix (blue) in the pellet area from OA treated MSCs (30%) when compared to untreated MSCs with only approximately 8% of the pellet area stained blue (Fig. 4B). Notably, the increase of bone matrix in BMP2 treated MSCs was further enhanced by OA treatment indicating a synergistic effect occurs when both OA and BMP2 are used. To further confirm this enhanced bone matrix formation, we performed IHC for OPN (Fig. 4A). Our results clearly showed a significant increased expression of OPN in OA and BMP treated cell pellets suggesting more osteoblastic cells in this group.

## Discussion

Fractures and bone defects are common in both military and civilian populations with over 2.2 million bone repair surgeries worldwide each year^26^. This bone repair process is regulated by multiple molecular cascades involves type I collagen matrix production and the participation of many signaling molecules, including ALP, Runx2, OC, and OPN^27^. Despite fractures being such a common problem, there is no approved drug that speeds up bone healing, or increases the chance of proper repair in clinic. In this regard, developing injectable drug or certain molecules that promotes fracture repair and prevents non-union is increasingly receiving attention.

Oleanolic acid, a natural triterpenoid extract from various type of plants has drawn increased attentions due to its effect of anti-inflammation, antivirus, hepatoprotection, and antitumor. Previously, OA, or its derivative, was demonstrated to activate the JNK pathway both in normal cells and malignant cells, as well as the suppression of mTOR pathway^28^. As these signaling pathways plays important role in mesenchymal stem cell differentiation, we further evaluated the effect of OA on MSC differentiation toward osteogenic cell lineage. After seven days of culture in osteogenic induction medium, a significantly enhanced ALP staining in OA treated MSCs demonstrated that OA could be used to induce stem cell osteogenesis *in vitro*. To look for possible molecules that involved in this process, several osteogenic marker genes were quantified by RT-PCR. Surprisingly, only osteogenic early stage markers (ALP, Runx2, Col1a1), not later stage markers (OC,OPN), were significantly induced by OA, indicating OA plays more important role in the onset stage of osteogenesis. Since our research group was the first to demonstrate inhibition of Notch signaling promotes onset differentiation of mesenchymal stem cell toward osteogenic cell lineage^20^, we next examined the effect of OA on Notch signaling in MSCs. As expected, during OA induced MSC osteogenic differentiation, Notch signaling was significantly inhibited in a dose-dependent manner suggesting a possible regulation of Notch signaling by OA in MSCs. To further understand the importance of Notch signaling in OA induced MSC osteogenic differentiation, we overexpressed NICD1 to activate Notch signaling in MSCs. The results clearly show that constitutive Notch activation fully blocked OA induced MSC osteogenesis, indicating inhibition of Notch signaling is required for OA induced MSC osteogenic differentiation.

To further investigate whether OA induced osteogenic differentiation is close to or comparable to BMP2 induced osteogenic differentiation, we performed ALP staining and RT-PCR in MSCs treated with either OA or BMP2, or both. Although our results showed a relative weak ALP staining in OA treated MSCs when compared to BMP2 treated MSCs, OA indeed greatly enhanced the BMP2 induced osteogenic differentiation in these cells. In order to further understand the possible molecular mechanism involved in OA potentiation of BMP2 action, we further investigated the effect of OA on phosphorylation of Smad1/5/8, a downstream target of BMPs that have been shown to directly stimulate bone formation and osteoblast differentiation^29, 30^. In the present study, OA treatment did not have a significant effect on the phosphorylation of Smad1/5/8 suggesting BMP signaling is not regulated by OA in MSC osteogenic differentiation. Although Notch signaling has been inhibited by OA, we have yet to identify the interaction between Notch signaling and BMP2-induced phosphorylation of Smad1/5/8. Further studies are still needed for determining the exact mechanism.

The combination of OA and BMP2 showed a more enhanced MSC osteogenic differentiation compared with each one alone *in vitro*, so we next performed ectopic bone formation analysis to validate this effect *in vivo*. After six weeks post-transplantation of OA treated MSCs under the dorsal skin, we observed a significantly improved osteogenesis in MSCs treated with OA and BMP2 when compared with each treatment alone. These consistent results from *in vitro* and *in vitro* further confirmed that OA has the ability to enhance osteogenesis and could also be used to promote BMP2 effect on bone tissue formation.

## Conclusion

**Figure 5:**
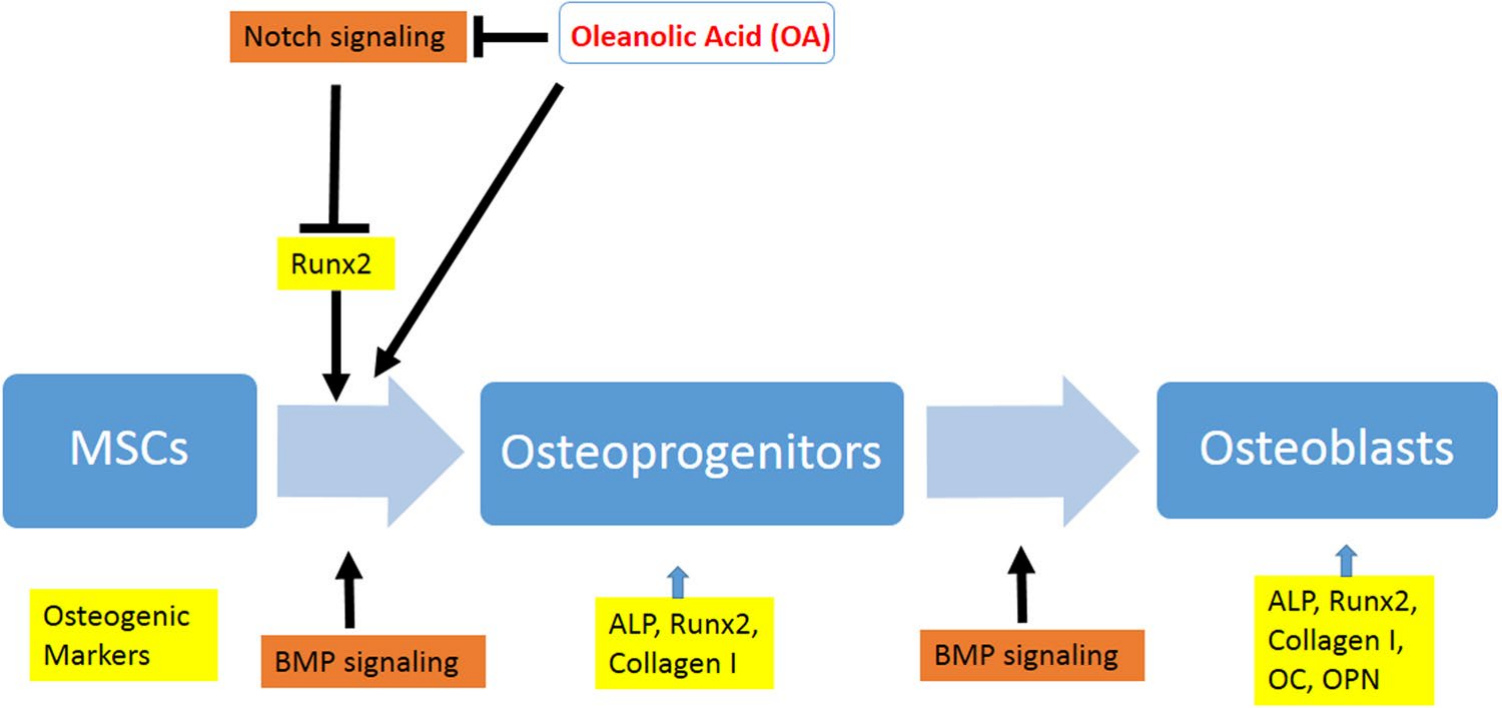
Possible model of OA in regulation of MSC osteogenic differentiation. Inhibition of Notch signaling by OA in MSC culture stimulates osteogenic gene expression (ALP, Runx2, Collagen I) that may lead to enhanced MSC osteogenic differentiation in the early stage of osteoblast formation. OA works parallel with BMP signaling to accelerate MSC differentiation toward to osteoblasts.

In summary, this study identified a natural extract from plants as a possible BMP2 substitute to promote bone tissue formation. Based on our results, we believe that OA can induce the onset of MSC osteogenic differentiation by inhibition of Notch signaling that leads to enhanced osteogenic gene expression (ALP, Runx2, Collagen I) and subsequent bone formation, which works parallel with BMP signaling to accelerate MSC differentiation toward to osteoblasts (Fig. 5). We hope that delivery of OA alone or in combination with BMP2 in different stages of fracture healing may lead to a better bone formation with an improved clinical safety profile. Further *in vivo* experiments will be needed to test the efficacy of OA on fracture and bone defect healing processes.

## Electronic supplementary material

Supplemental information
