# Supplementary material for: Oleanolic Acid Enhances Mesenchymal Stromal Cell Osteogenic Potential by Inhibition of Notch Signaling
Source: Sci Rep. 2017 Aug 1;7:7002. doi: 10.1038/s41598-017-07633-7 (PMC5539294; doi:10.1038/s41598-017-07633-7)

Oleanolic Acid Enhances Mesenchymal Stromal Cell Osteogenic Potential by Inhibition of Notch Signaling

Bing Shu[1], Yongjian Zhao[1], Yongjun Wang[1], Guangxi Wang[2], Xifu Shang[3], Michael Britt[2], Margaret Olmedo[2], Marjorie Chelly[2], Massimo Max Morandi[2], Shane Barton[2], Yufeng Dong[1, 2]*

1. Longhua Hospital and Key Laboratory of Ministry of Education of China, Shanghai University of Traditional Chinese Medicine, Shanghai, China.
2. Department of Orthopedic Surgery, LSU Health Sciences Center-Shreveport, LA USA.
3. Department of Orthopedic Surgery, Anhui Provincial Hospital, Hefei, Anhui, China

Running Title: Oleanolic acid enhances cell differentiation

* Corresponding Author:

Yufeng Dong, Ph.D, MD. Department of Orthopedic Surgery, LSU Health Sciences Center-Shreveport, LA USA. Email: ydong@lsuhsc.edu

Supplemental figure S1: Uncropped images for Western blots in this study.

(A) Full-length blots/gels of NICD1 in figure 2A. (B) Full-length blots/gels of β-actin in figure 2A.

(C) Full-length blots/gels of p-Smad1/5/8 in figure 3D. (D) Full-length blots/gels of β-actin in figure 3D.

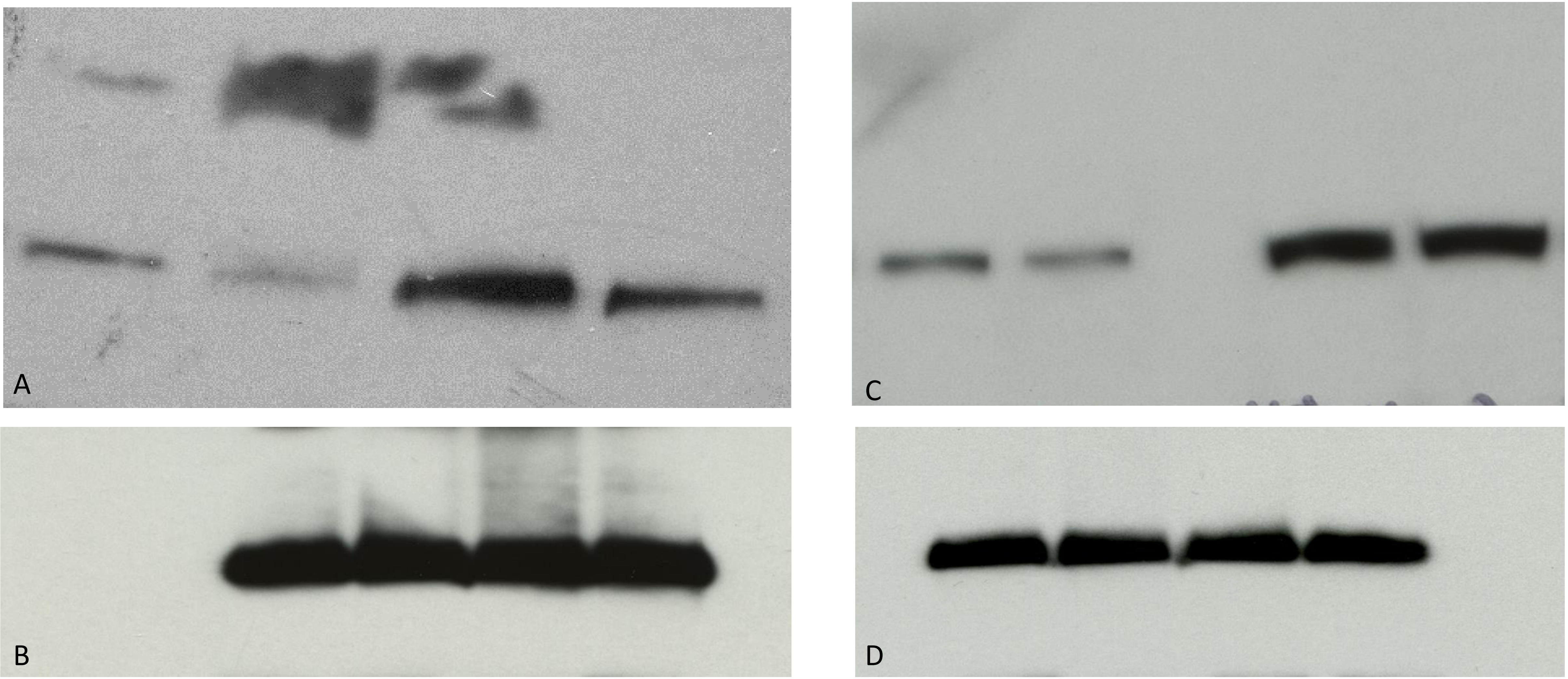

Supplement: Supplementary file 1 — Supplemental information [file 41598_2017_7633_MOESM1_ESM.pdf]
